# Conformational switching within dynamic oligomers underpins toxic gain-of-function by diabetes-associated amyloid

**DOI:** 10.1038/s41467-018-03651-9

**Published:** 2018-04-03

**Authors:** Melissa Birol, Sunil Kumar, Elizabeth Rhoades, Andrew D. Miranker

**Affiliations:** 1 Department of Chemistry, University of Pennsylvania, 231S. 34th St, Philadelphia, PA 19104 USA; 2 Department of Chemistry, New York University, Silver Center for Arts and Science, 100 Washington Square East, 10th Floor, New York, NY 10003 USA; 3 Department of Molecular Biophysics and Biochemistry, Department of Chemical and Environmental Engineering, Yale University, 260 Whitney Avenue, New Haven, CT 06520-8114 USA

## Abstract

Peptide mediated gain-of-toxic function is central to pathology in Alzheimer’s, Parkinson’s and diabetes. In each system, self-assembly into oligomers is observed and can also result in poration of artificial membranes. Structural requirements for poration and the relationship of structure to cytotoxicity is unaddressed. Here we focus on islet amyloid polypeptide (IAPP) mediated loss-of-insulin secreting cells in patients with diabetes. Newly developed methods enable structure-function enquiry to focus on intracellular oligomers composed of hundreds of IAPP. The key insights are that porating oligomers are internally dynamic, grow in discrete steps and are not canonical amyloid. Moreover, two classes of poration occur; an IAPP-specific ligand establishes that only one is cytotoxic. Toxic rescue occurs by stabilising non-toxic poration without displacing IAPP from mitochondria. These insights illuminate cytotoxic mechanism in diabetes and also provide a generalisable approach for enquiry applicable to other partially ordered protein assemblies.

## Abstract (web summary)

Toxic gain-of-function by islet amyloid polypeptide (IAPP) is thought to be mediated by membrane poration. Here the authors develop diluted-FRET to show that changes in pore structure correlate with onset of toxicity inside insulin secreting cells.

## Introduction

Advancing molecular insight faces unique challenges when structure-function arises from partially ordered protein oligomers^1^. Dynamic oligomers occur in aqueous and membrane milieus, for example, giving rise to nucleoli^2^ and signalling complexes^3^, respectively. As is often the case in biology, functional examples are a product of selective pressure optimising an intrinsic physical property of polypeptides. Unchecked, this property can also result in the misassembly of normally monodisperse proteins. Gains-of-function from these states include cytotoxicity while loss-of-function can occur as a result of irreversible fibre formation^4^.

Islet amyloid polypeptide (IAPP) is a 37-residue peptide-hormone cosecreted with insulin by the β-cells of the pancreas. In patients with type 2 diabetes, IAPP aggregation is associated with loss-of-β-cell mass^5^. Orthologues and mutational studies reveal a strong correlation between IAPP amyloid formation potential and cytotoxicity. Nevertheless, it is the structurally poorly defined, oligomeric species that are present prior to fibrillar aggregates that appear cytotoxic. In solution, IAPP weakly samples α-helical conformations that are stabilised upon interaction with phospholipid bilayers^6^. Membrane catalysed self-assembly follows binding with gains-of-function that include energy independent membrane translocation, membrane poration and mitochondrial localisation^7^. At the molecular level, the relationships of structures to functions, including downstream observations of mitochondrial dysfunction and cell death, remain unclear.

Membrane interacting oligomers whose structures and dynamics result in poration are widely believed to underpin IAPP’s toxic gain-of-function^8^. Membrane poration has also been reported for Aβ from Alzheimer’s disease, α-Synuclein from Parkinson’s disease and PrP from spongiform encephalopathy. These proteins also contain disordered regions. Numerous studies using synthetic vesicles have provided evidence for many alternative mechanisms: discrete pore formation, membrane disruption through carpeting, removal of phospholipid like a surfactant or as response to the induction of surface tension^9^. Which, if any, of these mechanisms is relevant inside cells is unknown. The role of molecular structure in these phenomena is also unclear although there is indirect evidence, such as the near loss-of-poration upon truncation of the non-membrane bound and disordered C-terminal residues^10^. Additionally, as IAPP localises to mitochondria, it is an appealing, albeit unproven idea that IAPP uncouples mitochondria leading to several downstream dysfunctions^11^. Clearly a deeper understanding of the molecular basis of IAPP-mediated poration and its relationship to toxicity is required.

To address this challenge, we carried out parallel measurements of IAPP on membrane models and inside live cells. Synergy between cellular and model measurements was achieved, in part, by engineering a permeation assay that uses a minimally processed, cell derived membrane. Using this assay, two sequentially sampled forms of poration are newly identified. A previously developed IAPP-specific ligand, ADM-116, is used here as a tool and shows toxic mitochondrial depolarisation to be associated with only one of the two forms of poration. A structure-based fluorescence approach, which we term diluted-FRET, is developed that brings focus onto large, homooligomeric assemblies of IAPP. We find that functional oligomers contain scores to hundreds of IAPP, populate several discrete states and none of these states are canonical amyloid. Our results support a model whereby IAPP-mediated toxicity derives from large, mitochondrial membrane associated assemblies of IAPP.

## Results

### IAPP mediates two classes of membrane permeation

**Fig. 1:**
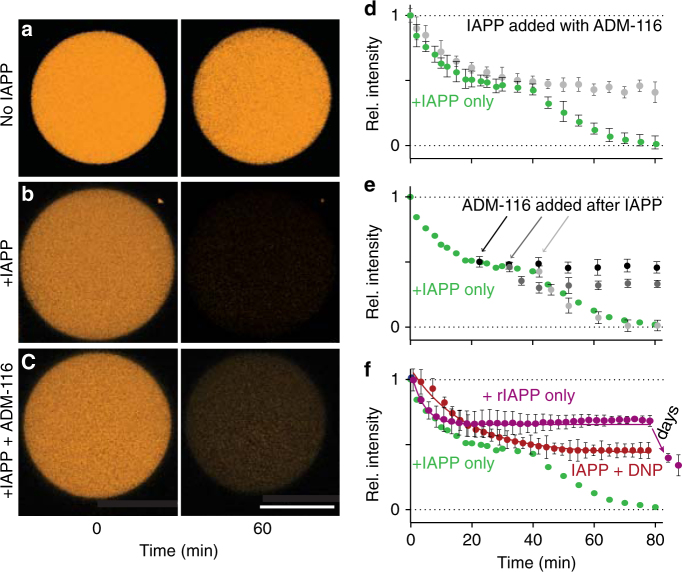
IAPP induces poration in a cell derived membrane. **a**–**c** Representative confocal images of CellTracker Orange labelled GPMVs at the indicated time points. Shown are single GPMVs observed without added IAPP **a**, IAPP added to a ratio of 10:1 (IAPP:lipid), **b** and IAPP added in the presence of ADM-116 to a ratio of 10:1:10 (IAPP:lipid:ADM-116), **c**. **d** Integrated intensities across repeats of data as in **a**–**c** used to determine kinetic profiles of leakage from 5 μM (phospholipid monomer units) GPMVs. **e** Leakage profile at 50 μM IAPP in the presence of equimolar ADM-116 added at the indicated delayed time points. **f** Leakage profiles upon addition of 10 μM DNP or 200 μM of the sequence variant of IAPP from rats, rIAPP. Confidence intervals are standard deviations from pairs of experiments conducted on three separate occasions, i.e., *n* = 6. Corresponding data for human "+IAPP only" in **e** and **f** are shown as an overlay without confidence intervals. Scale bar for all images is 20 μm

IAPP induces two kinetic phases of leakage in giant plasma membrane vesicles (GPMVs). GPMVs were prepared from live INS-1 cells as previously described^12,13^ with an additional step of pre-staining cytoplasmic components with a thiol-reactive fluorescent probe, CellTracker Orange (Fig. 1a). Upon addition of monomeric (Supplementary Fig. 1) 50 μM IAPP to 5 μM GPMVs, a protein:lipid (P:L) ratio of 10:1, two phases of leakage are observed (Fig. 1b, d). This P:L is consistent with addition of low-μM protein to cell culture which can result in P:Ls of 100:1 or more^14^. Moreover, high-local concentrations of IAPP are expected in vivo as IAPP is a secreted protein. The first phase of leakage is exponential, *k* = 33 ± 2 s^−1^, ending at a plateau in which only 48 ± 4% of the starting intensity is lost. A second exponential decay, *k* = 49 ± 2 s^−1^, begins after 1400 ± 200 s. This lag but not the rates are strongly sensitive to protein concentration (Supplementary Fig. 2a). The separation of two nearly identical rates by a lag phase suggests that leakage is an indirect reporter of two separate poration phenomena.

**Fig. 2:**
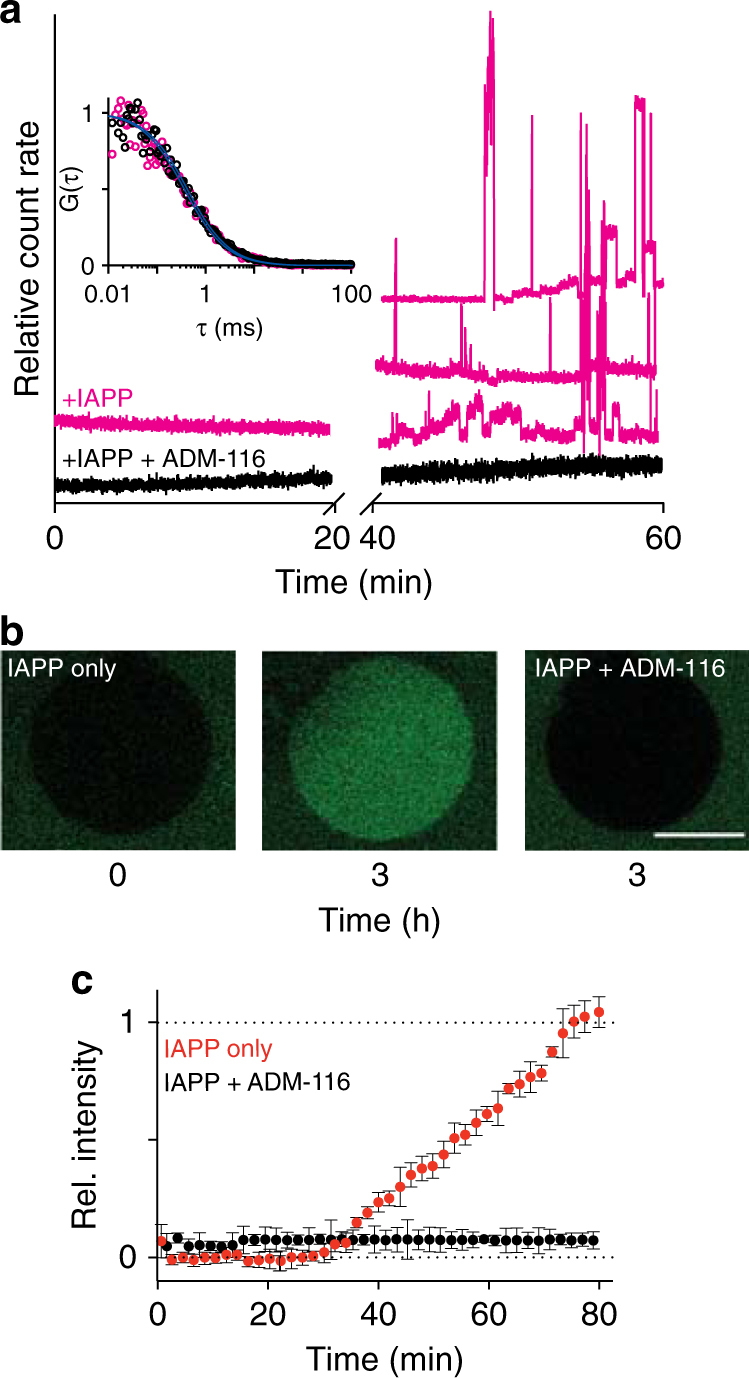
Alternative sieve sizes resulting from IAPP oligomer mediated poration. **a** FCS of fluorescent conjugates that have leaked out of 5 μM lumen labelled GPMVs after application of 50 μM IAPP. Representative traces of photon bursts are monitored over time in the absence (magenta) and presence of equimolar ADM-116 (black). Conditions are matched to those used in Fig. 1b and c. Inset: two representative autocorrelation analyses from the first 20 min overlaid with fits (blue) to a single-diffusing species. Autocorrelations were similarly computed for traces taken from 40 to 60 min (Supplementary Fig. 3). **b** Confocal fluorescence imaging of 500 nM GFP in the presence of 5 μM unlabelled GPMVs. Leakage was initiated by applying 50 μM IAPP. Conditions are matched to Fig. 1b, c. A representative 0 and 3 h time point of the same GPMV is shown. On right is a representative 3 h time point in which 50 μM ADM-116 was also present. **c** Quantitation of kinetics of data as in **b** using summed intensities of GFP across the equatorial plane of GPMVs. Confidence intervals are standard deviations taken from a minimum of nine repeats. Scale bar for all images is 20 μm

The two classes of IAPP-induced membrane permeation have distinct sieve size. Fluorescence correlation spectroscopy (FCS) was used to assess the diffusion of probes that escaped the lumen of the GPMVs. In the absence of added IAPP, only background counts are present. During the first kinetic phase, autocorrelation analysis is possible and fits are readily made to an analytical form that includes a single-diffusing species (Fig. 2a and inset). This gives a diffusion constant of 390 ± 25 μm^2^/s which corresponds in size to unconjugated dye. In marked contrast, data collection during the second phase of leakage is characterised by large irregular bursts that can persist for seconds and longer (Fig. 2a and Supplementary Fig. 3). This reflects the presence of much larger species (10–100 s of kDa)^15^. Clearly, the two phases of leakage are characterised by a transition from one in which only small and consistently sized holes are present to one in which large heterogeneously sized species can escape.

Poration by IAPP is bidirectional. To measure influx, poration was monitored by application of GFP (26 kDa) to a solution of unlabelled GPMVs. The exclusion of GFP is readily observed and stable (Fig. 2b). Application of 50 μM IAPP at 10:1 P:L shows exclusion of GFP persisting for the first ~30 min. This is followed by an abrupt change in which fluorescence within the lumen rises to that of the surrounding buffer within ~80 min (Fig. 2c). Exclusion during the first 30 min is consistent with large molecule efflux also not occurring on this timescale (Fig. 2a). Small sieve size poration is also bidirectional as evidenced using a small molecule fluorescence quencher (Supplementary Fig. 2b). Clearly, there is a discrete transition between small and large sieve size poration regardless of whether this is observed using influx or efflux assays.

### Leakage resulting in large sieve size can be inhibited

ADM-116 is a recently developed^16,17^ small molecule inhibitor of IAPP-induced cellular toxicity (Supplementary Fig. 7) (*P* = 0.0001). ADM-116 has been shown to bind specifically to α-helical, membrane-bound conformations of human IAPP. Moreover, ADM-116 can cross the plasma membrane and has been shown to directly bind intracellular IAPP. It is therefore uniquely suited to making measurements on GPMVs that can be paired with assessments performed inside live cells (see below).

IAPP assemblies associated with first- and second phase leakage are structurally distinct. Leakage profiles were collected on GPMVs with the addition of equimolar ADM-116 (Fig. 1c,d). The resultant profile is exponential, *k* = 34 ± 1 s^−1^, with an amplitude of 52 ± 3 %. This is within error of the first phase of leakage observed in the absence of compound (Fig. 1d). FCS of the media shows that only small solutes, with a diffusion coefficient of 480 ± 50 μm^2^/s, have been released (Fig. 2a and Supplementary Fig. 3). In measurements using GFP as the reporter, equimolar ADM-116 robustly inhibits influx into the GPMV lumen (Fig. 2b, c). In summary, this ligand shows no effect on small sieve-size leakage processes and wholly inhibits formation of large sieve states. As ADM-116 binding to IAPP is sequence and conformation specific^16,17^, this suggests that structurally distinct assemblies mediate the two forms of observed leakage.

ADM-116 is specific to pre-lag phase states of IAPP. Experiments were performed in which ADM-116 was added at early, middle and end stages of the lag phase (22, 32 and 42 min, respectively). At 22 min, the results are indistinguishable from co-addition of the compound at *t* = 0 (Fig. 1e). By 42 min, ADM-116 no longer affects the kinetics or sieve size of membrane poration. Partial effects to kinetics and sieve size are apparent for additions of compound at 32 min. These observations reinforce the interpretation that the transition from one leakage phase to next is a result of a structural change to membrane associated IAPP.

### Poration occurs in transient bursts

**Fig. 3:**
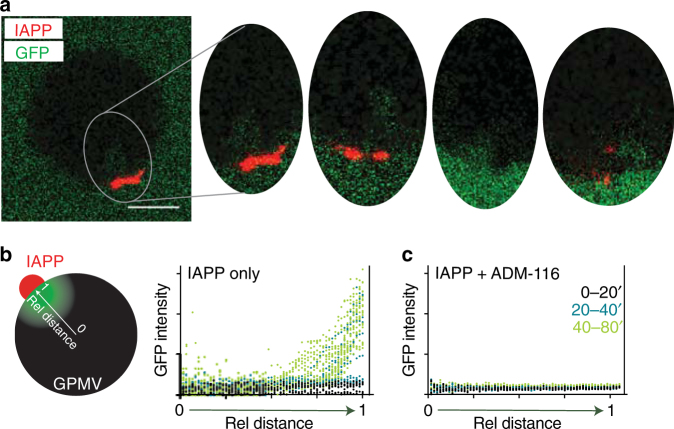
Poration occurs in discrete bursts at the site of IAPP oligomers. **a** Confocal fluorescence imaging of unlabelled GPMVs incubated in GFP containing buffer. Data are collected under conditions matched to Fig. 2b with addition of 200 nM IAPP labelled with Alexa 594. Entry of GFP into the lumen of GPMVs can be seen to occur as discrete bursts near IAPP oligomers (red). Four representative ROIs from independent GPMVs are shown. Note, for the right two ROIs, IAPP is more abundantly present in an adjacent *z*-slice. Scale bar for is 20 μm. **b**, **c** Kinetic evaluation of plume formation near IAPP oligomers. The intensity of GFP fluorescence along a radial line, (indicated schematically by an arrow that ends at an IAPP oligomer) was determined in the absence (**b**) or presence (**c**) of equimolar ADM-116

Time resolved imaging was performed using two probes: GFP and 50 μM IAPP doped with 200 nM IAPP labelled with Alexa 594. Striking, transient plumes of GFP can be seen localised to IAPP puncta (Fig. 3a). Many time points can be simultaneously considered by integrating intensity along an axis from the center of the GPMV to the IAPP puncta (Fig. 3b). In the first 20′, no GFP influx is observed. After 20′, plumes are readily apparent and grow more regular in frequency. Each GPMV has many IAPP puncta resulting in the observed global increase in intensity over time (Fig. 3b). Similar measurements monitoring efflux also shows unambiguous localisation of leakage to IAPP puncta (Supplementary Fig. 4). Importantly, when influx measures are performed in the presence of equimolar ADM-116, IAPP puncta are present but plumes of GFP do not occur (Fig. 3c). Previous work from our group suggested that large membrane bound oligomers convert between open and closed states in response to nucleation initiated by a subset of IAPP within the oligomer^18^. We speculate that such nucleation could form the basis of gating which in this assay, manifests as transient plumes of GFP.

The sequential sampling of two forms of poration is a specific property of IAPP. We determined that 10 μM of the mitochondrial protonophore, 2,4-dinitrophenol (DNP)^19^, can induce CellTracker Orange efflux from GPMVs at a rate of 58 ± 8 s^−1^ (Fig. 1f). Intensity loss plateaus at ~50% with no large species released (Supplementary Fig. 5) and material leaked has a diffusion constant of 420 ± 24 μm^2^/s. These measures are within error of the first kinetic phase observed using IAPP. Importantly, there is no evidence of a second phase. In a contrasting study, a sequence variant of IAPP was used. Rat IAPP (rIAPP) is generally presented as non-toxic as cytotoxicity requires protein concentrations that are significantly elevated compared to human IAPP^20^. Here, 200 μM of rIAPP was empirically determined to induce a first-phase leakage rate comparable to human IAPP (Fig. 1f). The kinetic profile that follows is qualitatively similar in that a plateau is present that is followed by a second phase of leakage. Strong quantitative differences are present. This includes a higher plateau, a lag phase of 10 s of hours and a second leakage phase that does not go to baseline. Plainly, IAPP has the capacity to induce two forms of leakage and the properties that govern these are dependent on sequence.

### IAPP alters intracellular membrane integrity

**Fig. 4:**
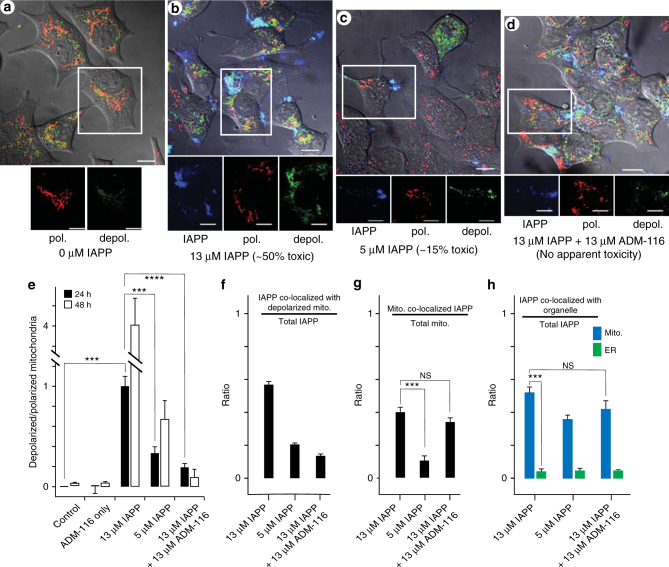
Mitochondria co-localised IAPP is associated with depolarisation. **a** Representative INS-1 cells stained with JC-1 marker. Two fluorescence channels in red and green correspond to polarised and depolarised mitochondria, respectively. These are shown and merged with the differential interference contrast (DIC) image. A region of interest (ROI) is chosen to show channels individually. **b** Representative cells exposed to toxic (13 μM) concentrations of IAPP doped with 200 nM IAPP_A647_ for imaging. Cells were stained with JC-1 marker 24 h after IAPP. The three channels, IAPP_A647_ (blue), polarised (red) and depolarised (green) mitochrondria are shown merged with the DIC image. A ROI is chosen to show channels individually. **c** As in **b** but at 5 μM IAPP. **d** As in **b**, but with the addition of 13 μM ADM-116 three hours after addition of IAPP (to ensure IAPP is intracellular^16^). **e** Image overlap statistics for **a**–**d** showing ratio of depolarised/polarised mitochondria at the indicated conditions and incubation time. **f** Image overlap statistics for **a**–**d** showing fraction of total IAPP overlapping depolarised mitochondria. **g** Image overlap statistics for **a**–**d** showing fraction of total mitochondria co-localised with IAPP. **h** Image statistics showing fraction of total IAPP overlapping with mitochondria and ER. Representative images for these statistics are shown for mitochondria (**b**–**d**) and ER (Supplementary Fig. 6), respectively. **e**–**h** For each measurement, a total of 50 cells from three biological replicates were selected for analysis. Error bars are standard deviations. Statistical significance of the data was calculated (Student’s *T*-test). Comparisons made in the main text are indicated here with lines and are annotated as ***P < 0.001 and ****P < 0.0001. *P*-values >0.01 are regarded as not significant (NS)

IAPP localisation to mitochondria is correlated with depolarisation. Mitochondrial polarisation in live INS-1 cells was measured using the fluorophore, JC-1^21^ (Fig. 4a). Roughly 50% of intracellular IAPP was localised to mitochondria (Fig. 4h). In contrast, only ~5% of available IAPP overlaps with endoplasmic reticulum (ER) (Fig. 4h and Supplementary Fig. 6) (*P* = 0.0003). At 13 μM IAPP, >50% cell death is apparent after 48 h (Supplementary Fig. 7). This is accompanied by widespread mitochondrial depolarisation (Fig. 4b, e). Imaging at 24 h, a time point before cell death is widespread, shows considerable depolarisation (Fig. 4e) relative to the no IAPP control (*P* = 0.002). At this point, more than half of the observable IAPP is localised to depolarised mitochondria (Fig. 4f). That is in the lead up to cell death, mitochondria that show co-localised IAPP also show depolarisation. Toxicity can be reduced by conducting studies at reduced concentrations of IAPP. At 5 μM IAPP, only ~15% of cells are dead after 48 h (Supplementary Fig. 7). Imaging under this reduced toxic condition at 24 h shows the fraction of mitochondria with co-localised IAPP (Fig. 4g) and depolarisation (Fig. 4e) are both greatly reduced (*P* = 0.002 and 0.001, respectively). Plainly, localisation of IAPP to mitochondria is associated with depolarisation and the extent of depolarisation correlates with toxicity.

Cytotoxic rescue by ADM-116 eliminates mitochondrial depolarisation without displacing IAPP. Small molecule rescue was used as an orthogonal approach to reducing IAPP-induced toxicity. Addition of equimolar ADM-116, 3 h after uptake of 13 μM IAPP wholly rescues INS-1 cells from toxicity^16^. Depolarisation is markedly reduced compared to the 13 μM IAPP-only images (Fig. 4d, e) (*P* = 0.0001). Importantly, ADM-116 does not significantly change the fraction of IAPP co-localised to mitochondria (Fig. 4h) (*P* = 0.48), nor the fraction of mitochondria with associated IAPP (Fig. 4g) (*P* = 0.52). This suggests that toxicity is reduced instead by changing the structure of mitochondria associated IAPP. Our observation of mitochondrial depolarisation in response to IAPP may be direct or may be upstream of stimulating other cellular factors. For example, mitochondria initiated apoptosis includes membrane poration by Bcl-2 family proteins^22^. Whether direct or indirect, changes in mitochondria localised IAPP structure that we have associated with changes in poration sieve size appear central to the origins of toxicity.

### Oligomers are dynamic during membrane leakage

**Fig. 5:**
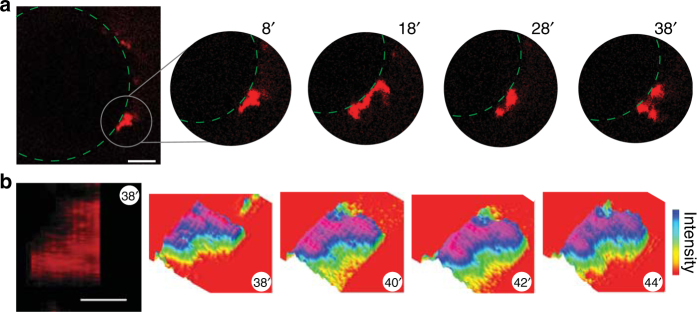
IAPP oligomers are dynamic. Confocal fluorescence imaging of 50 μM IAPP doped (500:1) with IAPP_A594_ and applied to 5 μM GPMV. Solution conditions are matched to those used for FRET studies (Fig. 8). **a** A representative *z*-slice of a GPMV showing a region of interest (ROI) that contains an oligomer. Imaging at 10′ intervals show evolution in oligomer structure. Green dashed lines indicate the location of the GPMV surface. **b** Three-dimensional reconstruction of one oligomer from a narrow ROI near the equatorial plane and several neighbouring *z*-planes. The reconstructed image has been rotated to show the oligomer from a perspective normal to the plane of the GPMV. Example of raw fluorescence is shown (left) followed by colour coded intensity over a series of four time points at 2′ intervals. Scale bar for all images is 20 μm

Starting with GPMVs and moving into cells, the above functional analyses were paired with tools for assessing the gross morphology of IAPP oligomers. Remarkably, fluorescently labelled IAPP puncta on GPMVs show time dependent changes (Fig. 5a, b). Oligomer growth dominates observation. Changes in oligomers that can be interpreted as localised losses in size, fission and/or fusion are also routine. These changes in IAPP oligomer morphology occur on minute timescales, which is comparable to the observed IAPP-induced leakage rates (Fig. 1). This suggests that IAPP dynamics and poration are coupled.

**Fig. 6:**
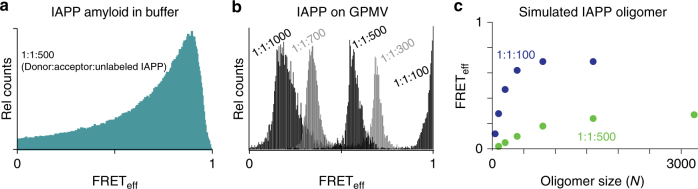
Observed and expected behaviour for diluted FRET in oligomers of IAPP. **a** Canonical amyloid fibres were prepared by dilution of a stock solution of IAPP into aqueous buffer. Stock solution contained a mixture of IAPP_A488_ (donor), IAPP_A647_ (acceptor) and unlabelled IAPP at 1:1:500. Confocal imaging was used to prepare a FRET_eff_ histogram (green). **b** Intermolecular FRET between IAPP_A488_ (donor) and IAPP_A647_ (acceptor) detected on GPMVs at the indicated ratios of donor:acceptor:unlabelled protein. Each condition contains a protein equivalent of ADM-116 to maintain a stable conformation (Fig. 7a, c). Each distribution represents the sum of observations taken from at least three different GPMVs. **c** Simulated average FRET_eff_ for a dynamic, isotropic spherical distribution of an oligomer composed of *N* copies of IAPP. Relevant simulation constants are volume per IAPP (4000 Å^3^), Förster distance (52 Å) and the indicated ratio of (donor labelled IAPP):(acceptor labelled IAPP):(unlabelled IAPP)

Changes in dynamic oligomer morphology are also evident using intermolecular FRET. IAPPs were prepared in which either a donor (Alexa 488, IAPP_A488_) or acceptor (Atto 647, IAPP_A647_) is covalently attached to the N-terminus. Unlabelled IAPP is then mixed in high proportion (>50:1) with labelled peptides. This orthogonal approach, which we term “diluted-FRET”, focuses data collection on large oligomers. Oligomers, with a monomer count (*N*) less than the dilution ratio, are statistically unlikely to have both donor and acceptor present. As a non-dynamic control, amyloid fibres of IAPP were prepared in aqueous buffer using 1:1:500 (donor:acceptor:unlabelled IAPP) and imaged using confocal microscopy. A broad distribution of FRET_eff_ is obtained (Fig. 6a). When donor and acceptor fluorophores change their orientations and/or positions on a timescale that is slow relative to the integration time of measurement (here, 0.5 s per pixel), then the expectation is that a broad distribution of FRET efficiencies will be observed, reflecting the underlying spatial distribution of donor–acceptor fluorophores^23^. This is observed here for canonical amyloid fibres, where the peptide components are not expected to have mobility. In marked contrast, the FRET efficiency distributions observed for the oligomers are very narrow (Fig. 6b); if the donor and acceptor peptides are rapidly equilibrating through multiple conformations on the timescale of the measurement, then narrow peaks corresponding to the population weighted average FRET efficiency are observed. As the FRET observed here is intermolecular, the narrow peaks reflect averaging from oligomer dynamics.

**Fig. 7:**
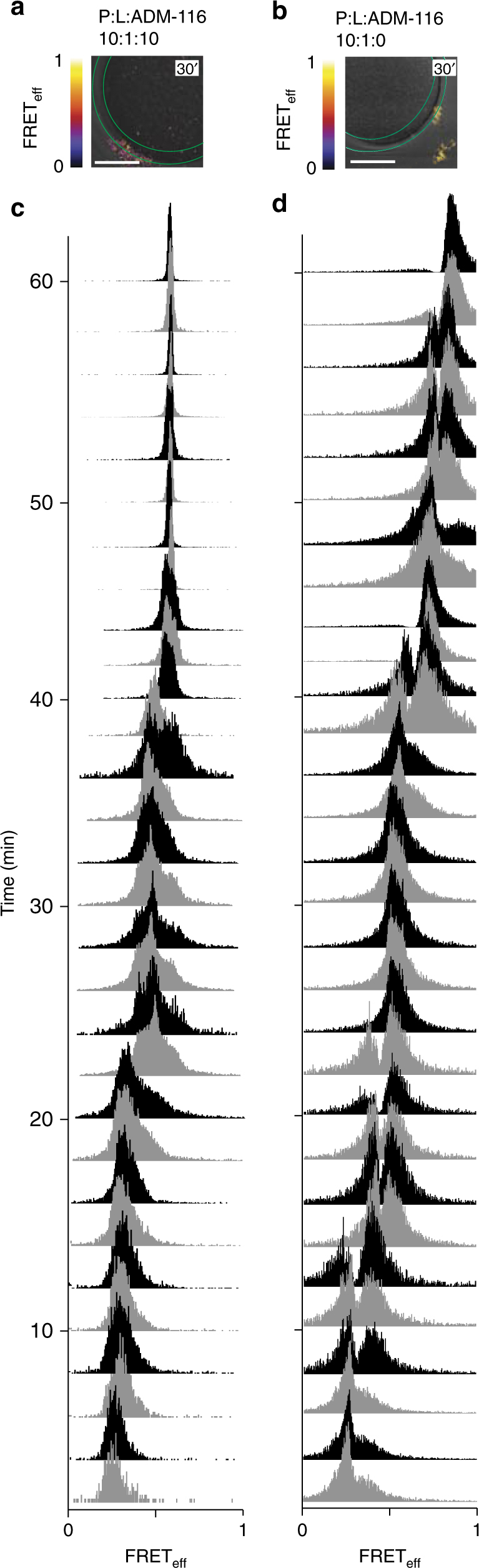
Time dependent changes within membrane localised IAPP oligomers. **a**, **b** Representative intermolecular FRET_eff_ histograms between IAPP_A488_ and IAPP_A647_ in the presence of 50 μM unlabelled IAPP (1:1:500) in the presence (**a**) and absence (**b**) of equimolar ADM-116. Confocal FRET images are shown as a heat map merged with corresponding DIC images. Ring shaped ROIs (green) are used to collect data that focus only on membrane associated IAPP such as are shown as histograms in **c**, **d**. Scale bar, 10 μm. **c**, d Data from single-optical sections of GPMVs monitored over time after exposure to IAPP and ADM-116 at ratios of 10:1:10 (**c**) and 10:1:0 (**d**) protein:lipid:ADM-116. Images were recorded every 2 min

**Fig. 8:**
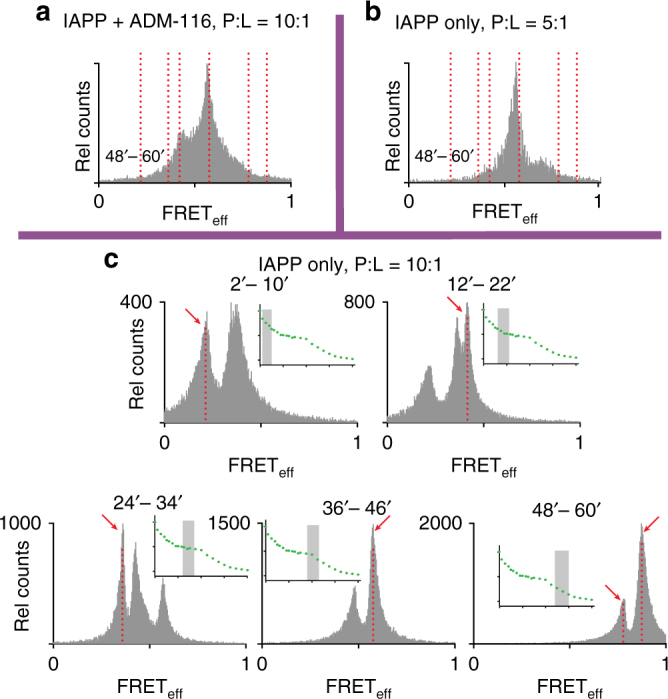
Structural changes within IAPP oligomers occur on the same timescale as poration. **a** Intermolecular FRET histograms at 10:1:10 IAPP:lipid:ADM-116 summed over a minimum of three GPMVs, five optical sections for each GPMV and the indicated periods of time. Conditions and confocal fluorescence data collection are matched to those shown in Fig. 7. **b** As in **a** but at 5:1:0 IAPP:lipid:ADM-116. **c** Diluted-FRET at 10:1:0 IAPP:lipid:ADM-116. Pixel statistics correspond to summation across the indicated time period. Red arrows indicate peaks used to define FRET_eff_ values for species referred to in the main text and the locations of dotted red lines in **b**. Inset: Schematic of leakage profile collected under matched conditions (Fig. 1d). Grey rectangles show the time period over which FRET_eff_ is summed in the associated histogram

GPMV associated IAPP forms a discrete species when in complex with ADM-116. Under conditions that display only small sieve size poration (P:L:ADM-116 = 10:1:10) (Fig. 1c, d), FRET_eff_ is readily measured from membrane associated puncta using 1:1:500 (donor:acceptor:unlabelled IAPP) (Fig. 7a). Time dependent changes to FRET_eff_ show a progression of transitions over ~40′ (Fig. 7c), i.e., structural transitions are occurring on the same timescale as leakage. Signal averaging across repeats gives a final profile of the stabilised state (Fig. 8a). The continued presence of relatively narrow peaks suggests that the small molecule stabilised species are still dynamic.

### Two poration modes mediated by distinct non-amyloid states

Under conditions for observing two phase leakage (P:L:ADM-116 = 10:1:0) (Fig. 8d), four peaks are apparent over the first 34′ with FRET_eff_ at 0.21 ± 0.07, 0.36 ± 0.04, 0.41 ± 0.08 and 0.58 ± 0.02, respectively (Fig. 8e). The first three FRET_eff_ peaks dominate over a period of time in which only small molecule leakage is observed. The second poration phase, 48′–60′, shows a marked shift to two high-FRET_eff_ ensembles at 0.78 ± 0.04 and 0.87 ± 0.01. The coincidence of the timescale of these transitions with that observed for leakage (where all IAPP is unlabelled) shows that fluorophore interactions are not contributing significantly to the structures sampled by IAPP oligomers. This is further confirmed by observation of comparable changes when using an alternate donor/acceptor pair (Supplementary Fig. 8a). None of these states yield FRET_eff_ or histological staining^24^ similar to amyloid (Fig. 6a). Moreover, reducing the protein concentration by half strongly lowers the sampling of second phase leakage on the 48′–60′ timescale (Supplementary Fig. 2a). The FRET_eff_ over this time does not show significant sampling near 0.78 and 0.87 (Fig. 8b) and indeed, more closely resembles what is observed using ADM-116 ligand during this phase of leakage (Figs. 7a, b and 8a). Clearly, membrane associated IAPP oligomers are internally dynamic and are not dominated by amyloid structures over the period of time where poration is observed. Moreover, both time dependence and inhibitor studies show different FRET_eff_ species to be associated with the two leakage processes.

IAPP oligomers grow by oligomer and not monomer addition. Inspection of individual FRET_eff_ time courses at 2′ resolution shows peaks change in intensity, but not position (Fig. 7c, d). That is new peaks appear as a result of others disappearing rather than shifting. This is most further apparent in the time averaged data (Fig. 8). The absence of significant peak broadening upon averaging indicates that peaks do not shift in position over the time course. That these changes likely represent oligomer growth is evident in the approximately fivefold increase in intensity over time (Fig. 8). These observations suggest that oligomer growth does not occur by monomer or even small (*N* < ~10) oligomer addition. Instead, the peaks correspond to distinct species the sheer number of which (>6) make it unlikely that they represent conformational transitions. Rather, we suggest that progression in FRET_eff_ includes contributions that are the result of a stepwise merging of large-*N* oligomers.

### Dynamic oligomers are responsible for cell toxicity

**Fig. 9:**
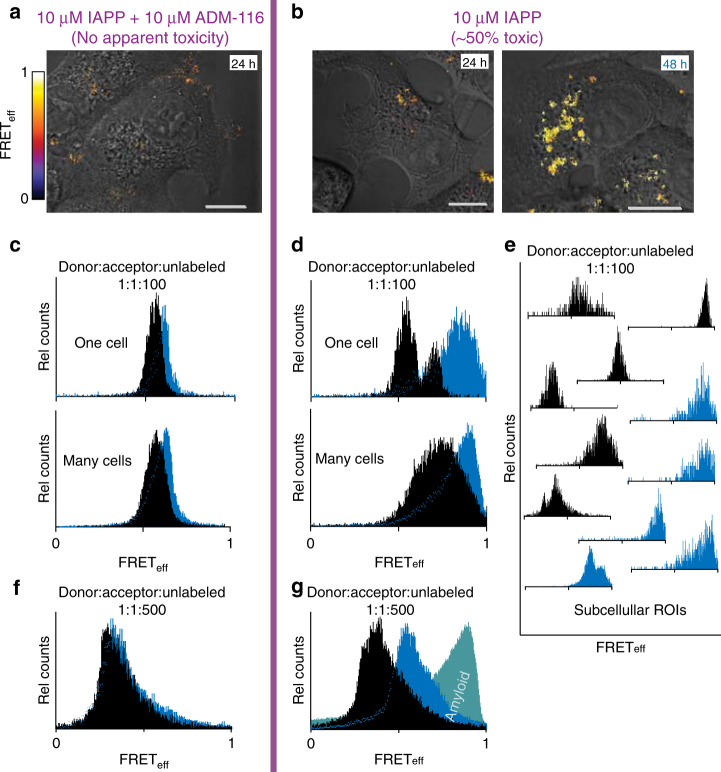
Intracellular oligomer conformations under toxic and rescued conditions are distinct. **a**, **b** Intermolecular diluted FRET between IAPP_A488_ and IAPP_A647_ (in the presence of unlabelled IAPP, 1:1:100) imaged in live INS-1 cells under rescued (**a**) and toxic (**b**) conditions. Pixel-based FRET analysis of representative cells is shown as a heat map merged with corresponding DIC images. Scale bar, 10 μm. **c**–**g** Histograms of pixel counts for FRET_eff_ images as in **a** and **b**. **c** FRET_eff_ histograms for cells at 24 h (black) and 48 h (cyan) for the ADM-116 rescued condition (**a**). Analysis for a representative single cell is shown above the sum of data collected from 50 cells from three independently conducted experiments on each of three different days. **d** Analysis as in **c**, but under conditions of ~50% toxicity (10 μM IAPP). **e** Analysis as in **d**, but for representative ROIs within single cells. **f**, **g** Effect of FRET dilution on experiments matched to **b** and **c** except that IAPP_A488_ and IAPP_A647_ are instead diluted 1:1:500 with 10 μM unlabelled IAPP. Sums of data collected from 50 cells are shown. **g** Intracellular FRET observed under toxic conditions are not amyloid. Diluted-FRET of the toxic condition, 10 μM IAPP, measured inside cells after 48 h (cyan). These are overlaid with FRET_eff_ statistics of a control in which IAPP is in an amyloid conformation at 1:1:500 (green, Fig. 6a)

Intracellular IAPP oligomers adopt a single-overall structure when toxicity is rescued. INS-1 cells were incubated with 10 μM IAPP and rescued from toxicity by addition of equimolar ADM-116 3 h later (our standard condition^16^ to ensure intracellular interaction). Intermolecular FRET is widespread indicating that large *N* oligomers of IAPP are abundant (Fig. 9a). The FRET_eff_ pixel statistics show a single-narrow peak with FRET_eff_ = 0.60 ± 0.07 (Fig. 9c). Over 48 h, this distribution does not significantly change (Fig. 9c). A repeat analysis under at a different dilution of the FRET pair, (1:1:500) (Fig. 9f), and analysis using a different FRET pair, IAPP_A488_ and IAPP_A594_, give similar results (Supplementary Fig. 9b, c), suggesting that fluorophore–fluorophore interactions do not contribute significantly to the observation. The doping ratio of 1:1:100 (donor:acceptor:unlabelled IAPP) that is predominantly used in our cell studies was determined empirically to give data with the best dynamic range. This compares to 1:1:500 used for GPMVs. This suggests that the intracellular oligomers are smaller than those observed on GPMVs. Importantly, a single, long-lived discrete assembly of intracellular IAPP is formed upon cytotoxic rescue by ADM-116; a result directly comparable with that observed on GPMVs (Figs. 7a, c and 8a).

Intracellular oligomers of IAPP evolve to high-FRET_eff_ under conditions that are cytotoxic. Diluted-FRET was measured at 24 h and 48 h as above, but without rescue using ADM-116 (Fig. 9b). Cumulative analysis of cell images taken at 24 h reveal a broad range of FRET_eff_ = >0.5 (Fig. 9d). Individual cells (Fig. 9d), as well as small sub-cellular ROIs (Fig. 9e) show recurring and relatively narrow distributions. This is in line with observation of discrete species observed on GPMVs (Figs. 7b, d and 8c). After 48 h, cells show significant morphological signs of dysfunction. This is accompanied by FRET distributions shifting to markedly higher efficiencies (Fig. 9d, e). Plainly, the evolution of IAPP oligomer conformations inside cells (and their inhibition with ADM-116) mimic those sampled on GPMVs.

**Fig. 10:**
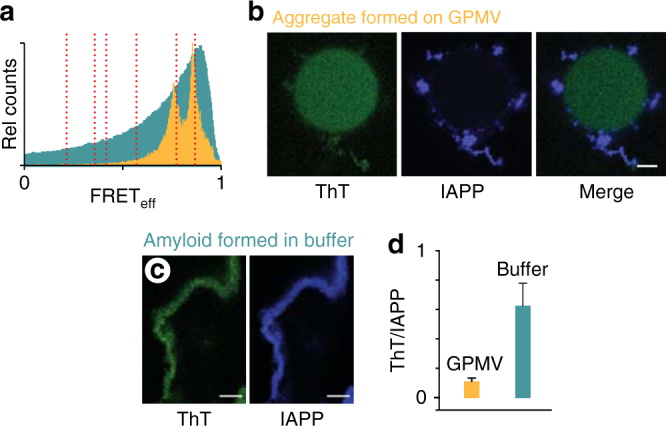
IAPP oligomers on GPMVs are not amyloid. **a** Intermolecular FRET_eff_ statistics from 50 ROIs of aggregates prepared using IAPP_A488_, IAPP_A647_ and unlabelled IAPP (1:1:500). GPMV induced aggregates (orange) were prepared under conditions of 10:1:0 IAPP:lipid:ADM-116 with images taken 60′ after exposure of 50 μM IAPP to 5 μM GPMV. These are overlaid with FRET_eff_ statistics of a control in which IAPP is in an amyloid conformation at 1:1:500 (Fig. 6a). Confocal imaging and FRET_eff_ analysis was performed using procedures identical to that used for the GPMVs. Vertical dotted red lines correspond to specie positions defined in Fig. 8e. **b**, **c** Confocal imaging of IAPP aggregates prepared from IAPP doped with IAPP_A647_ (500:1) and further stained with the amyloid indicator dye, ThT. Two channel image pairs are shown for ThT (green) and IAPP_A647_ (blue), respectively. Aggregates were prepared either in the presence of GPMVs (**b**) or in buffer (**c**). Scale bar for all images is 5 μm. **d** Image statistics for data as in **b**, **c**. Presented ThT intensities normalised to IAPP_A647_ intensities using ROIs away from the GPMV surface. Error bars are standard deviations from sets of experiments conducted on three independent occasions

Intracellular oligomers of IAPP associated with cytotoxicity are not canonical amyloid fibres. Diluted-FRET experiments were repeated in cells using a doping ratio matched to that used for the GPMV studies above, 1:1:500 (donor:acceptor:unlabelled IAPP). In the absence of small molecule, the FRET_eff_ progresses with peaks at 0.36 ± 0.08 and 0.62 ± 0.04 at 24 h and 48 h, respectively (Fig. 9g). The FRET_eff_ distributions measured under these toxic conditions are clearly not the same as that of amyloid fibres prepared in aqueous buffer (Figs. 9g and 10).

## Discussion

The use of GPMVs as model system enabled the identification of two forms of IAPP-mediated membrane permeation. This is apparent from two kinetic phases in the leakage profiles (Fig. 1d) each with a characteristic size distribution for the escaping molecules (Fig. 2). Small molecule binding to IAPP inhibits the second, but not the first, phase of leakage (Fig. 1d). A parallel can be drawn between these results and the mitochondrial depolarisation measurements in cells (Fig. 4), where the small molecule ligand reverses the depolarisation seen in the presence of IAPP, without displacing IAPP from mitochondria. This observation is significant, as it shows localisation of IAPP to mitochondria to be necessary but not sufficient for toxicity. Instead, it appears that the structure of IAPP at the mitochondria is a more relevant indicator of toxic effect. We propose that nucleated conversion of small pore permeable states to large pore permeation is the origin of gains-in-toxic function by IAPP. This is an important distinction as membrane poration need not be cytotoxic.

The transition between small and large pore states reflects a change in oligomer topology, and/or the conformation of IAPP within the oligomer. This is suggested by the deviations of observed FRET_eff_ from simple models. For example, isotropic packing of IAPP into a sphere at high density (4000 Å^3^/IAPP) can be simulated. At the doping ratios used in GPMV experiments, 1:1:500, FRET_eff_ cannot exceed ~0.27 (Fig. 6c). This is clearly smaller than all but one of the observed peaks (Fig. 8c). Additionally, a ligand stabilising a membrane bound α-helical form of IAPP affects only one of the two phases of poration (Fig. 1). Taken together, these results support our assertion that there are distinct membrane associated oligomeric states.

The evolution of the amyloid-hypothesis into the oligomer-hypothesis was pioneered, in part, by the immunostained light-microscopy observation of membrane associated puncta^25^. A recent effort with bearing on our work is a study in which SDS was used as a membrane model^26^. Toxic IAPP oligomers, chromatographically stable, discrete in size (~90 kDa), rich in α-helical secondary structure, shown to enter cells and localise to mitochondria were reported. Remarkably, anti-sera from patients with diabetes but not healthy controls were cross-reactive with this oligomer. The 90 kDa size and subcellular localisation observed in that construct are consistent with the size and localisation observations reported here. Our current study does not exclude a significant role for dimer through hexamer formation as suggested by others^27–29^. Instead, our work clarifies that oligomers associated with gains-in-toxic function are much larger and internally dynamic.

In functional systems, dynamic assemblies of membrane proteins have been observed for Nephrin^30^ and more recently LAT^3^, which forms micron sized clusters during T-cell activation. The multistate nature of dynamic IAPP we report here is unlikely to be unique. If functional membrane systems also leverage disordered regions to create alternative properties, their transitions between states would likely be under regulatory control. We have demonstrated here using IAPP that such transitions are sufficiently defined as to be addressable with a protein-specific ligand. The same may therefore be true for functional dynamic protein phase transitions.

## Methods

### Materials

Chemicals were purchased from Sigma Aldrich (St. Louis, MO) unless otherwise specified. Thioflavin T (ThT) was purchased from Acros Organics (Fair Lawn, NJ) and islet amyloid polypeptide (IAPP) from Elim Biopharmaceuticals (Hayward, CA, USA). ADM-116 was previously prepared as described^16^. IAPP stocks were prepared by solubilising ~2 mg protein in 7 M guanidinium hydrochloride. The solution was filtered (0.2 micron) and transferred to C-18 spin column, washed twice with water followed by a wash of 10% acetonitrile, 0.1% formic acid (v/v) and then eluted into 200 µL of 50% acetonitrile, 0.1% formic acid (v/v). The concentration of IAPP was determined by OD at 280 nm (*ε* = 1400 M^−1^ cm^−1^). The solution was then divided into single use aliquots (20–50 µL), lyophilised, and stored at −80 °C. Stock solutions of IAPP were prepared with water from these aliquots.

Alexa 488 carboxylic acid succinimidyl ester (A488), Atto 647 N succinimidyl ester (A647) and Alexa 594 succinimidyl ester (A594) dyes were purchased from Life Technologies (Carlsbad, CA). IAPP labelling was prepared as described previously^20^. Briefly, IAPP was incubated with dye, succinimidyl ester on a MacroSpin column for 4 h. Labelled IAPP was eluted from the MacroSpin column with 50% acetonitrile/0.2% formic acid solution. This was then diluted with 7 M guanidinium hydrochloride solution to a total organic content of <5%. Labelled protein was then purified by reverse-phase high-performance liquid chromatography, and identity was confirmed by mass spectrometry. Aliquots were lyophilised and stored at −80 °C. Stocks at 100 μM in water were prepared and immediately before use. IAPP control fibres were prepared by incubating 50 μM hIAPP and 100 nM IAPP_A647_ in 50 mM sodium phosphate buffer, 100 mM KCl, pH 7.4, for ∼24 h. Fibres were pelleted at 21,000 g for 30 min and resuspended three times using water.

### GPMV

GPMVs were isolated from INS-1 cells as previously described^13^. Briefly, cells were plated in 35 mm dishes and cultured for 48 h, washed with a 10 mM HEPES, 150 mM NaCl, 2 mM CaCl_2_ (pH = 7.4) twice and then exposed to 2 mM N-ethyl maleimide (NEM, Sigma Aldrich, St. Louis, MI, USA) for 2 h. Collected samples were then passed over a gravity-flow column (Bio-Rad) containing size exclusion Sephacryl of pore size 400-HR (Sigma Aldrich, St. Louis, MI, USA) to separate GPMVs from residual cell debris. For leakage assays, the thiol-reactive fluorescent probe, CellTracker Orange (Thermo Scientific, Rochester, NY, USA) was first applied to cells at 1:1000 dilution and incubated for 1 h at 37 °C. The phospholipid content of unlabelled and labelled final material was measured by total phosphate assay. For leakage assays monitoring the influx of GFP, recombinant expressed GFP (500 nM) was added to the GPMV containing solution and the increase in fluorescence inside the GPMV was monitored.

### GPMV imaging

Images were obtained in 8-well NUNC chambers (Thermo Scientific, Rochester, NY, USA) containing 250 µl of GMPV at 5 μM phospholipid lipid concentration. Imaging was carried out at the Yale Department of Molecular, Cellular, and Developmental Biology imaging facility, on a Zeiss LSM 510 confocal microscope, using a ×63 Plan-Apo/1.4-NA oil-immersion objective with DIC capability (Carl Zeiss, Oberkochen, Germany). Image acquisition and processing were achieved using Zeiss Efficient Navigation (ZEN) and Image J software^31^.

### Fluorescence correlation spectroscopy (FCS)

FCS measurements were made on an LSM 880 Airyscan system NLO/FCS Confocal microscope (Carl Zeiss, Oberkochen, Germany) with a C-Apochromat ×40/1.2 NA UV-VIS-IR Korr. water immersion objective (Carl Zeiss, Oberkochen, Germany). Thiol-conjugated molecules were excited at 594 nm. Confocal pinhole diameter was adjusted to 70 μm. Emission signals were detected through a 607-nm long-pass filter. Measurements were made in 8-well chambered coverglasses (Nunc, Rochester, NY, USA). All samples were incubated in GPMV buffer (10 mM HEPES, 150 mM NaCl, 2 mM CaCl_2_ (pH = 7.4)) for 1 h prior to taking measurements. Autocorrelation data were collected at regular intervals (5 min) with each autocorrelation curve collected over 10 s with 30 repeats.

Autocorrelations were fit using the software QuickFit3.0^32^. For thiol-conjugates, a model for a single-diffusing species undergoing 3D Brownian diffusion was used.

1 $$G(\tau ) = \frac{1}{{N}} \times \left[ {1 + \frac{\tau }{{\tau _{d,1}}}} \right]^{ - 1} \times \left[ {1 + \frac{\tau }{{s^2\tau _{d,1}}}} \right]^{ - \frac{1}{2}},$$

here, *N* is the total thiol-conjugated molecules in the detection volume. The characteristic translational diffusion time of a diffusing particle is given by τ_*d,1*_. The structure factor, *s*, was determined as a free parameter for solutions of free Alexa 594 hydrazide dye and then fixed to the experimentally determined value of 0.1 for all subsequent fittings. For experiments of conjugates eluting during the second decay phase of leakage diffusion was assessed by burst counts within the time frame analysed.

### Confocal microscopy and cell imaging

Images were obtained in 8-well NUNC chambers (Thermo Scientific, Rochester, NY, USA) seeded with 20000–25000 cells/well. Cells were cultured for 48 h after passage before beginning experiments. For time dependent co-localisation experiments of IAPP with JC-1 mitochondrial staining, the medium contained 200 nM IAPP_A647_, 13 µM unlabelled peptide. For experiments in the presence of ADM-116, 13 µM of molecule was introduced in the medium following a 3 h incubation of cells with IAPP. For experiments monitoring mitochondria, JC-1 was incubated with cells at 1:5000, at 37 °C for 45 min prior to addition of protein. Images were acquired after 48 h total incubation time. Imaging was carried out using a ×100 Plan-Apo/1.4-NA oil-immersion objective with DIC capability (Carl Zeiss, Oberkochen, Germany). For all experiments reporting on the co-localisation of labelled IAPP, the gain setting for the blue channel was kept constant from sample to sample. Mitochondria containing JC-1 aggregates were detected in red (excitation 540 nm, emission 570 nm), and monomers in the green channel (excitation 490 nm, emission 520 nm). Image acquisition and processing were achieved using Zeiss Efficient Navigation (ZEN) and Image J software^31^.

### Intracellular imaging Förster resonance energy transfer

The INS-1 growth media was replaced with media containing 100 nM IAPP_A488_ and 100 nM IAPP_A647_ (or 100 nM IAPP_A594_) and unlabelled IAPP and incubated for the timescales indicated in the text. Media were replaced with fresh prior to imaging. In experiments where ADM-116 was used, small molecule was added 3 h after incubation with protein. Images were acquired using a ×100 Plan-Apo/1.4-NA oil-immersion objective with DIC capability (Carl Zeiss, Oberkochen, Germany). For the donor channel, IAPP_A488_ was excited with a 488 nm Argon2 laser and detected through a 505/550 nm emission filter. For the acceptor channel, IAPP_A647_ was excited with a 633 Argon2 laser and detected through a 730/750-nm emission filter. For all experiments the pinhole was kept constant to the *Z*-slice thickness of each filter channel. Single-cell images were obtained for donor alone, acceptor alone and donor–acceptor fusion channels. Image acquisition and processing were achieved using Zeiss Efficient Navigation (ZEN) and Image J software^33^. The Image J plugin, RiFRET^34^, was used to calculate and remove the bleed through for each channel and to calculate a pixel-based FRET efficiency. The FRET distance was then calculated using:

2 $$E = \frac{{R_0^6}}{{R_0^6 + r^6}},$$

where *E* is the calculated efficiency of FRET energy transfer, *R*_0_ is the Förster distance and *r* is the distance between the donor and the acceptor.

### Imaging FRET

GPMV experiments were conducted in 8-well NUNC chambers (Thermo Scientific, Rochester, NY, USA) including 250 µl of GMPV stock solution at 5 μM apparent phospholipid (in monomer units). Wells containing GPMVs were mixed with 100 nM IAPP_A488_ and 100 nM IAPP_A647_ (or 100 nM IAPP_A594_) and unlabelled IAPP. In experiments where ADM-116 was used, small molecule was added at the same time as protein. The same microscope setup and analysis procedure was used to image FRET in GPMVs and cells.

### Cell culture

Rat insulinoma INS-1 cells (832/13, Dr. Gary W. Cline, Department of Internal Medicine, Yale University) were cultured at 37 °C and 5% CO_2_ in phenol red free RPMI 1640 media supplemented with 10% fetal bovine serum, 1% penicillin/streptomycin (Life Technologies, Carlsbad, CA, USA), and 2% INS-1 stock solution (500 mM HEPES, 100 mM l-glutamine, 100 mM sodium pyruvate and 2.5 mM β-mercaptoethanol). Cells were passaged upon reaching ~95% confluence (0.25% Trypsin-EDTA, Life Technologies), propagated, and/or used in experiments. Cells used in experiments were pelleted and resuspended in fresh media with no Trypsin-EDTA. We follow ATCC guidelines for authentication of non-human cell-lines (technical bulletin #8, http://atcc.org). This includes monitoring mycoplasma, morphology and growth rate. We regularly thaw fresh stocks and begin and track new sequences of passages. We do not perform species verification.

### Cell viability

Cell viability was measured colourimetrically using the Cell-Titer Blue (CTB, Promega, Madison, WI, USA) fluorescence-based assay. Cells were plated at a density 5000 cells/well in 96-well plates (BD Biosciences, San Diego, CA). Peptide was directly introduced to each well after 48 h of culture and then incubated for an additional 48 h. For time dependent experiments, cells were incubated with peptide for the specified time points. After the incubation period, 20 µL CTB reagent was added to each well and incubated at 37 °C and 5% CO_2_ for 2.5–3.5 h. Fluorescence of the resorufin product was measured on a FluoDia T70 fluorescence plate reader (Photon Technology International, Birmingham, NJ, USA). All wells included the same amount of water to account for different concentrations of peptide added to sample wells. Wells that included water vehicle but not peptide served as the negative control (0% toxic), and wells containing 10% DMSO were the positive control (100% toxic). Percent toxicity was calculated using the following equation:

3 $${\mathrm{\% }}{\rm Toxicity} = 100 - \left[ {100 \times \left( {\frac{{ < S > - < P > }}{{ < N > - < P > }}} \right)} \right].$$

Each independent variable is the average of eight plate replicates from the negative control (<*N>*), positive control (<*P*>) and samples (<*S*>). Results presented for viability experiments are an average of three such experiments conducted independently on different days. Error bars represent the standard error of the mean.

### Phosphate assay

Lipid concentrations for GPMV preparations were determined by measuring total phosphate^35^, assuming that all measured phosphate is from phospholipids, and that all lipids are phospholipids. This is a practical assumption designed to ensure reproducibility.

### Simulation of diluted-FRET

Expected values of FRET_eff_ for dynamic, isotropic distributions of spherically distributed IAPP were computed by numerical integration coded in-house using Mathematica (Wolfram Research, Champaign, IL). Briefly, the experimental ratio of labelled to unlabelled protein defines a binomial probability for the number of fluorophores distributed within trial spheres. The size of the spheres was dictated by constants (stated in the main text) for protein density and the total number of placed protein molecules. Spherical oligomers were generated randomly until the cumulative FRET_eff_ converged to a single value. Occurrences of multiple acceptors and donors within a single oligomer were accommodated by treating each potential dye pair, *ij*, as having an independent probability of resonance transfer, *p*_*ij*_. For example, a trial oligomer containing a single donor and three acceptors located a distance of *R*_o_ away gives a FRET_eff_ = 0.875. Effects of donor–donor and acceptor–acceptor interactions were not considered. In general, multi-fluorophore corrections contribute negligibly to computations performed at the experimental ratios used in this work (Fig. 6c).

The fraction of oligomers of size N with one donor and one acceptor at a doping ratio of 1:1:X is binomial with a probability of (2/(X + 2)). This is further scaled by 0.5 to reflect the frequency that the two labelled IAPPs within the oligomer are donor and acceptor. Higher degrees of labelling within oligomers are rare at the doping ratios used in this work and so are ignored. To calculate the number of acceptor molecules contributing to the FRET signal detected, the number of pixels showing FRET was divided by the total number of acceptor pixels obtained from the acceptor channel.

### Statistical analysis

For each experiment, means and standard deviations (specified in Figure Legends) of parameters measured were determined. Statistical analyses were performed using the Student’s *t*-test and expressed as *p*-values in the text. All analyses were carried out with GraphPad Prism.

### Data availability

The data supporting the findings of this manuscript are available from the corresponding authors upon reasonable request.

## Electronic supplementary material

Supplementary Information(DOCX 20481 kb)

## References

[1] Courchaine EM, Lu A, Neugebauer KM (2016). Droplet organelles?. EMBO J..

[2] Brangwynne CP, Mitchison TJ, Hyman AA (2011). Active liquid-like behavior of nucleoli determines their size and shape in Xenopus laevis oocytes. Proc. Natl Acad. Sci. USA.

[3] Su X (2016). Phase separation of signaling molecules promotes T cell receptor signal transduction. Science.

[4] Aguzzi A, Altmeyer M (2016). Phase separation: linking cellular compartmentalization to disease. Trends Cell Biol..

[5] Mukherjee AMSD, Butler PC, Soto C (2015). Type 2 diabetes as a protein misfolding disease. Trends Mol. Med..

[6] Williamson JA, Loria JP, Miranker AD (2009). Helix stabilization precedes aqueous and bilayer-catalyzed fiber formation in islet amyloid polypeptide. J. Mol. Biol..

[7] Costes S, Langen R, Gurlo T, Matveyenko AV, Butler P (2013). C. beta-Cell failure in type 2 diabetes: a case of asking too much of too few?. Diabetes.

[8] Mukherjee A, Morales-Scheihing D, Butler PC, Soto C (2015). Type 2 diabetes as a protein misfolding disease. Trends Mol. Med..

[9] Last NB, Schlamadinger DE, Miranker AD (2013). A common landscape for membrane-active peptides. Protein Sci..

[10] Brender JR, Salamekh S, Ramamoorthy A (2012). Membrane disruption and early events in the aggregation of the diabetes related peptide IAPP from a molecular perspective. Acc. Chem. Res..

[11] Kegulian NC (2015). Membrane curvature-sensing and curvature-inducing activity of islet amyloid polypeptide and its implications for membrane disruption. J. Biol. Chem..

[12] Sezgin E (2012). Elucidating membrane structure and protein behavior using giant plasma membrane vesicles. Nat. Protoc..

[13] Schlamadinger DE, Miranker AD (2014). Fiber-dependent and -independent toxicity of islet amyloid polypeptide. Biophys. J..

[14] Wimley WC (2010). Describing the mechanism of antimicrobial peptide action with the interfacial activity model. ACS Chem. Biol..

[15] Elbaum-Garfinkle S, Ramlall T, Rhoades E (2010). The role of the lipid bilayer in tau aggregation. Biophys. J..

[16] Kumar S (2016). Foldamer-mediated manipulation of a pre-amyloid toxin. Nat. Commun..

[17] Kumar S, Birol M, Miranker AD (2016). Foldamer scaffolds suggest distinct structures are associated with alternative gains-of-function in a preamyloid toxin. Chem. Commun..

[18] Last NB, Rhoades E, Miranker AD (2011). Islet amyloid polypeptide demonstrates a persistent capacity to disrupt membrane integrity. Proc. Natl Acad. Sci. USA.

[19] Perry RJ, Zhang D, Zhang XM, Boyer JL, Shulman GI (2015). Controlled-release mitochondrial protonophore reverses diabetes and steatohepatitis in rats. Science.

[20] Magzoub M, Miranker AD (2012). Concentration-dependent transitions govern the subcellular localization of islet amyloid polypeptide. FASEB J..

[21] Perelman A (2012). JC-1: alternative excitation wavelengths facilitate mitochondrial membrane potential cytometry. Cell Death Dis..

[22] Vaux DL, Korsmeyer SJ (1999). Cell death in development. Cell.

[23] Schuler B, Lipman EA, Eaton WA (2002). Probing the free-energy surface for protein folding with single-molecule fluorescence spectroscopy. Nature.

[24] Wolfe LS (2010). Protein-induced photophysical changes to the amyloid indicator dye thioflavin T. Proc. Natl Acad. Sci. USA.

[25] Gurlo T (2010). Evidence for proteotoxicity in beta cells in type 2 diabetes: toxic islet amyloid polypeptide oligomers form intracellularly in the secretory pathway. Am. J. Pathol..

[26] Bram Y (2014). Apoptosis induced by islet amyloid polypeptide soluble oligomers is neutralized by diabetes-associated specific antibodies. Sci. Rep..

[27] Young LM, Cao P, Raleigh DP, Ashcroft AE, Radford SE (2014). Ion mobility spectrometry-mass spectrometry defines the oligomeric intermediates in amylin amyloid formation and the mode of action of inhibitors. J. Am. Chem. Soc..

[28] Abedini A., et al. Time-resolved studies define the nature of toxic IAPP intermediates, providing insight for anti-amyloidosis therapeutics. *Elife* **5**, 12977 (2016). 10.7554/eLife.12977 PMC4940161 27213520

[29] Nath A, Miranker AD, Rhoades E (2011). A membrane-bound antiparallel dimer of rat islet amyloid polypeptide. Angew. Chem. Int. Ed. Engl..

[30] Banjade S., Rosen M. K. Phase transitions of multivalent proteins can promote clustering of membrane receptors. *Elife* **3**, e04123 (2014). 10.7554/eLife.04123 PMC4238058 25321392

[31] Schneider CA, Rasband WS, Eliceiri KW (2012). NIH Image to ImageJ: 25 years of image analysis. Nat. Methods.

[32] J. W. Krieger J. L. QuickFit 3.0: a data evaluation application for biophysics. In: http://www.dkfz.de/Macromol/quickfit/ (2015).

[33] Soty MVM, Soriano S, del Carmen Carmona M, Nadal Aacute, Novials A (2011). Involvement of ATP-sensitive potassium (KATP) channels in the loss of beta-cell function induced by human islet amyloid polypeptide. J. Biol. Chem..

[34] Roszik J, Lisboa D, Szollosi J, Vereb G (2009). Evaluation of intensity-based ratiometric FRET in image cytometry--approaches and a software solution. Cytom. A.

[35] King EJ (1932). The colorimetric determination of phosphorus. Biochem. J..

