# Supplementary material for: Conformational switching within dynamic oligomers underpins toxic gain-of-function by diabetes-associated amyloid
Source: Nat Commun. 2018 Apr 3;9:1312. doi: 10.1038/s41467-018-03651-9 (PMC5882805; doi:10.1038/s41467-018-03651-9)
Supplement: Supplementary file 1 — Supplementary Information(DOCX 20481 kb) [file 41467_2018_3651_MOESM1_ESM.docx]

**
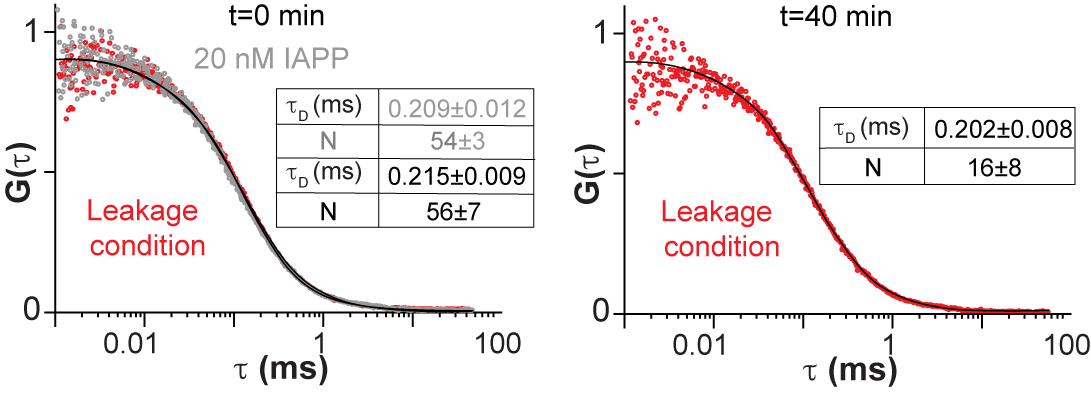
**

**Supplementary Figure 1 IAPP is monodisperse and monomeric in GPMV buffer.** We prepare IAPP in single use, freeze dried aliquots stored at -80C. These are first solubilized in pure water followed by dilution of the protein into buffer. This procedure routinely results in monomer peptide^1^. In this work, this is confirmed by FCS in which the confocal volume placed in buffer away from the GPMVs. Fits to obtain diffusion times require only a single diffusing species the mobility of which is consistent with previous observations of monomeric IAPP.

**
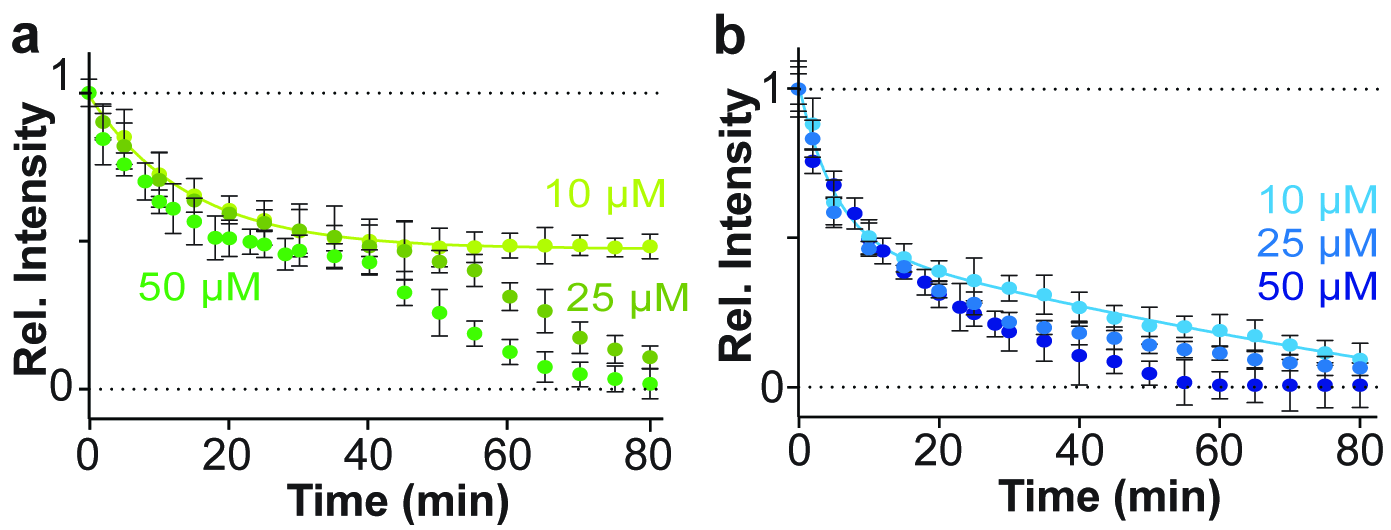
**

**Supplementary Figure 2 IAPP induced leakage is concentration dependent and bidirectional.**
**a** Integration of GPMV intensities across repeats of data as in Fig. 1 after addition of the indicated concentrations of IAPP. **b** As in **a**, but in the presence of 1 mM AB9 (a small molecule fluorescence quencher). Confidence intervals are standard deviations from pairs of experiments conducted on three separate occasions, i.e. n=6.


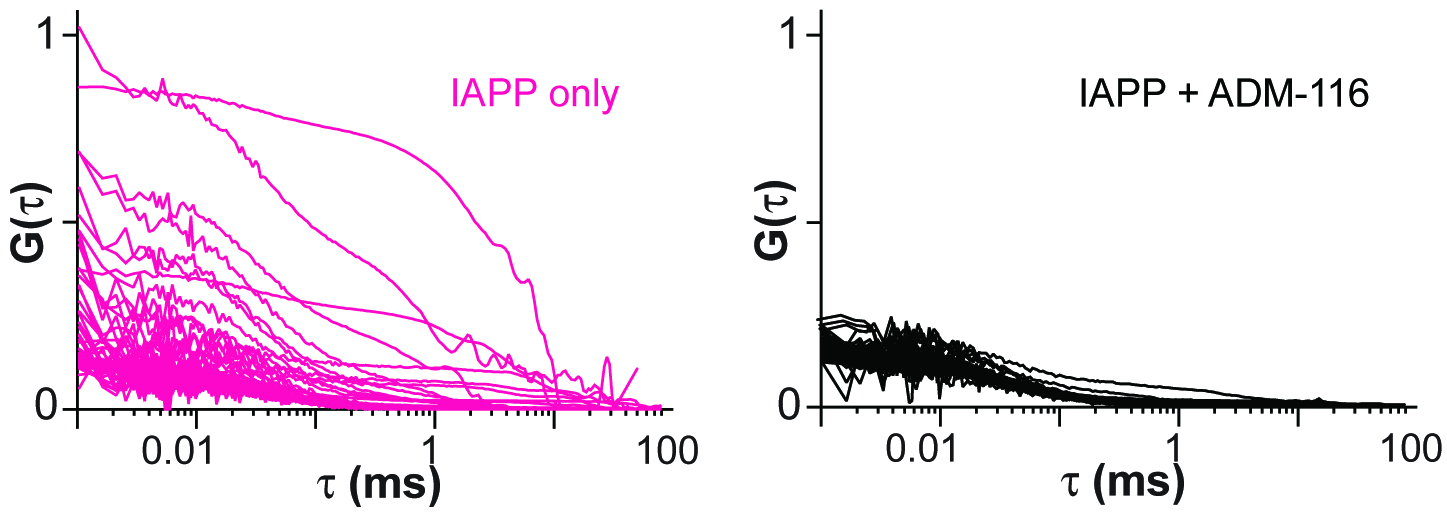


**Supplementary Figure 3 IAPP mediated poration sieve size monitored by FCS**. Representative autocorrelation curves from FCS of fluorescent conjugates that have leaked out of 5 μM lumen labelled GPMVs after application of 50 μM IAPP. Shown are data taken in the absence (magenta) and presence (black) of equimolar ADM-116. Representative traces of photon bursts for this data are also shown (Fig. 2a).

**
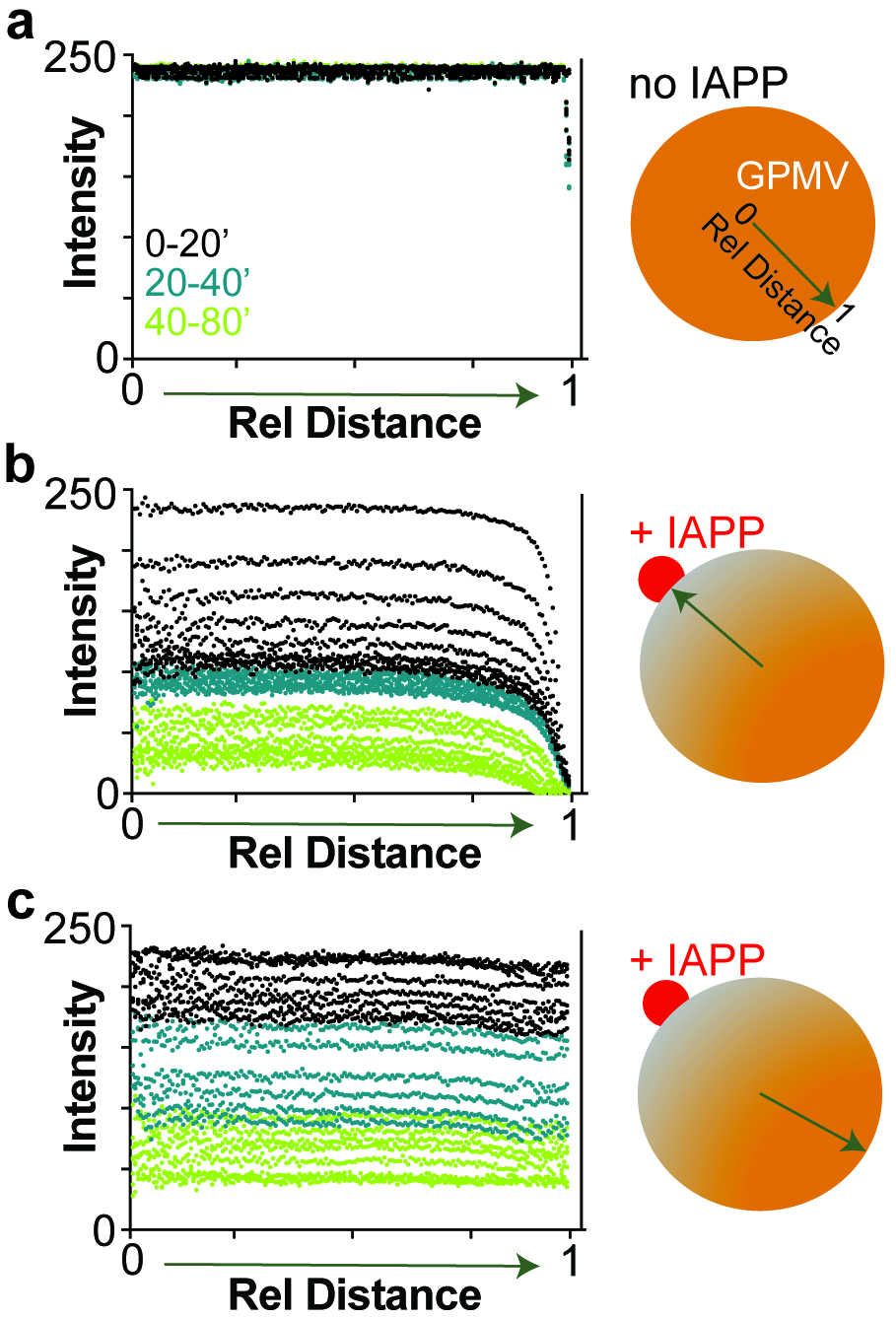
**

**Supplementary Figure 4 Lumen efflux occurs proximal to IAPP oligomers**. Radial analysis of cell tracker orange labelled GPMVs (Fig. 1). The intensity along a radial line (indicated by an arrow) was determined as a function of time in the absence (**a)** and presence (**b** and **c)** of 50 μM IAPP. Quantitation is shown for radii that terminate near to (**b)**, or away from (**c)** an IAPP oligomer.


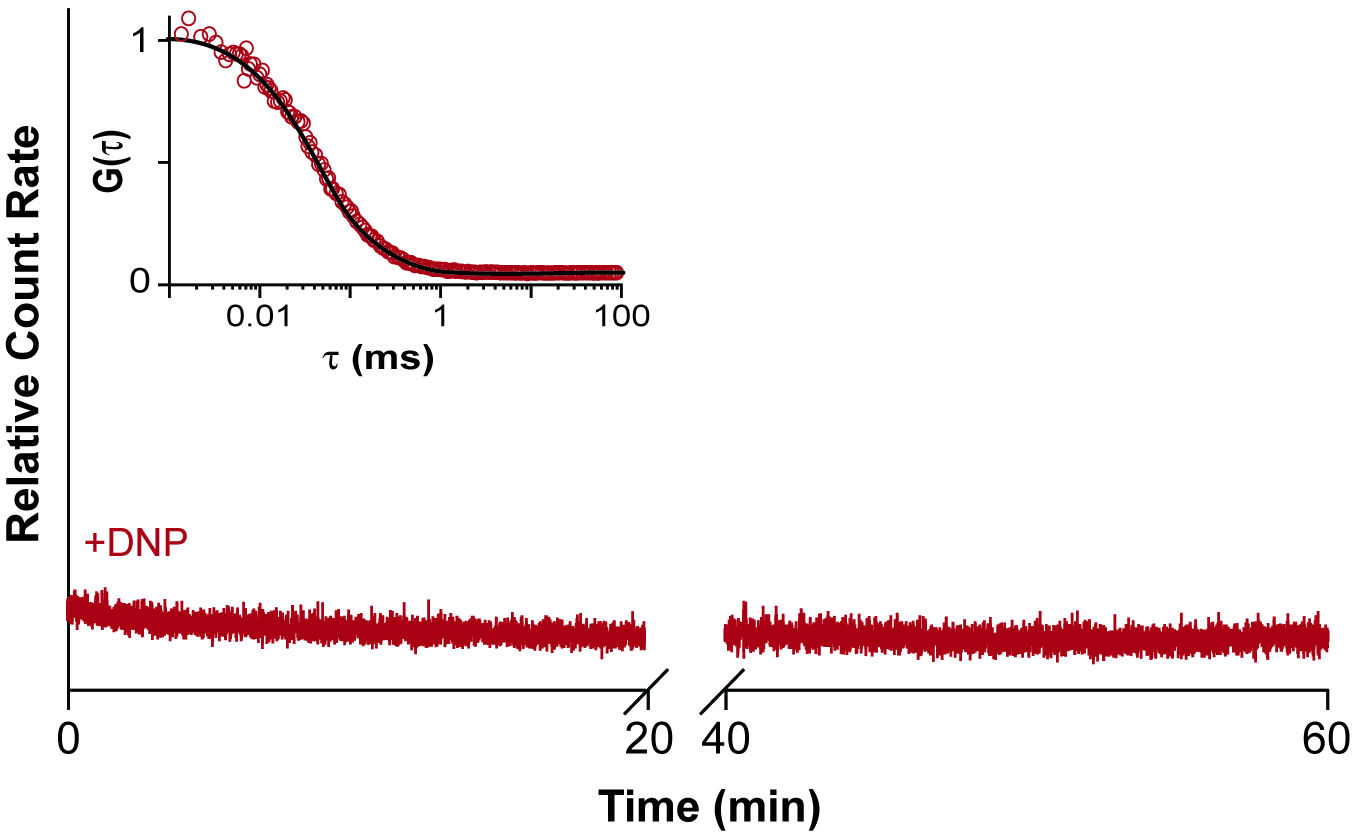


**Supplementary Figure 5 DNP mediated poration monitored by FCS**. Representative photon bursts from fluorophore labelled materials exiting GPMVs after addition of 10 μM DNP. **Inset**: autocorrelation of the last 20 minutes of data acquisition overlaid with a fit that includes only a single diffusing species.


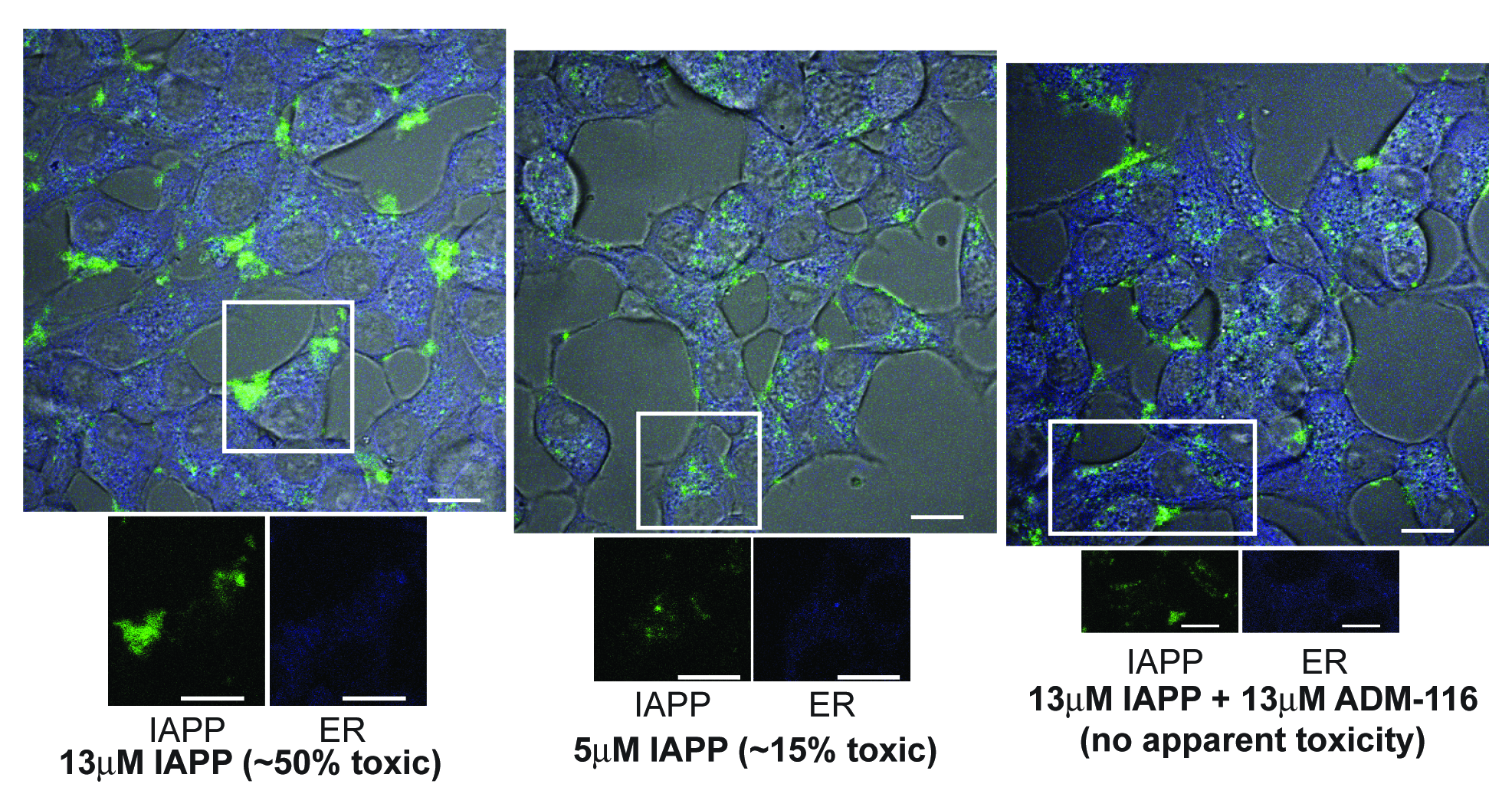


**Supplementary Figure 6 IAPP shows weak localisation with ER.** Representative INS‑1 cells stained with ER-Tracker. Fluorescence channels in green and blue correspond to IAPP_A488_ and ER marker respectively. These are shown merged with the DIC image. A region of interest (ROI) is chosen to show channels individually for each indicated condition. Cells were stained with ER-tracker 24 h after addition of IAPP. For each condition, image statistics from 50 cells, over three biological replicates were collected and are presented in the main text (Fig. 4h). Scale bar for all images is 20 μm.


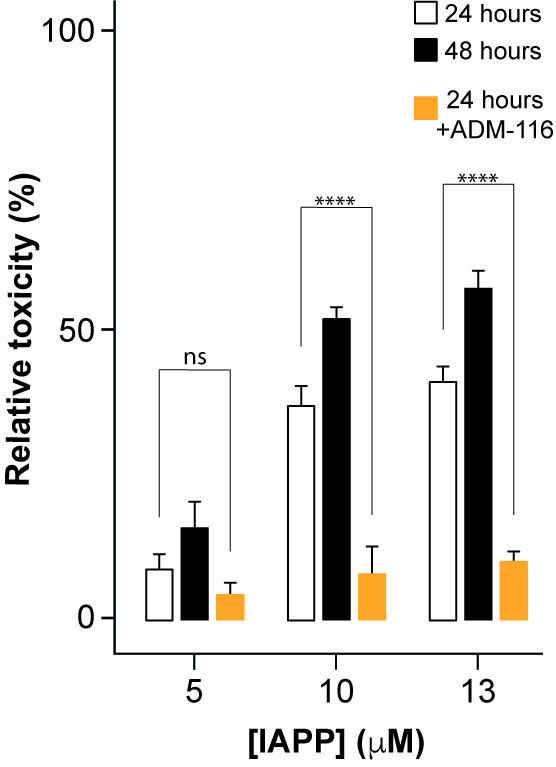


**Supplementary Figure 7 Cytotoxicity of IAPP**. Colourimetric measure of toxicity 24 and 48 h after introduction of the indicated concentration of human IAPP to INS‑1 cell culture. Data are expressed relative to vehicle-only addition prepared on the same well-plates. Measures of toxicity at 24 h were also conducted in the presence of equimolar ADM-116 confirming previously reported rescue from IAPP induced toxicity^2, 3^. Each histogram bar is the average of eight, on-plate repeats across each of three independently performed replicates (n = 24). Statistical significance of the data was calculated (Student’s t-test). Comparisons made in the main text are indicated here with lines and are annotated with **** corresponding to P<0.0001. P-values >0.01 are regarded as not significant (ns).


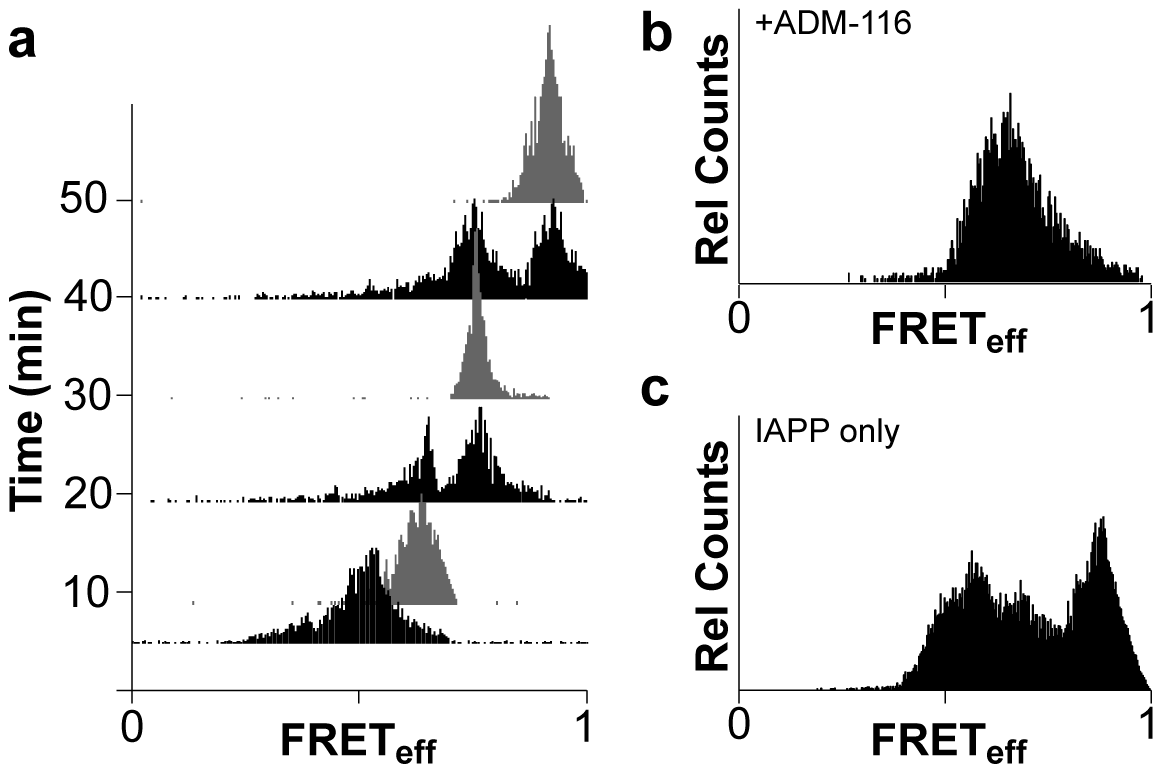


**Supplementary Figure 8 IAPP oligomer formation measured with an alternative dye pair**. **a** Under conditions matched to those used for Fig. 8, intermolecular FRET measured from images of GPMVs under conditions matched to Fig. 8b, but using IAPP_A488_ and IAPP_A594_ as the donor/acceptor pair. Representative histograms are shown from a single optical section of a single GPMV as a function of time. **b** Intermolecular FRET was measured 24 h after exposing INS-1 cells to 13 μM IAPP and equimolar ADM-116. IAPP was doped with 0.1 μM each of IAPP_A488_ and IAPP_A594_. **c** As in **b**, but without addition of ADM-116. For **b** and **c**, histograms are cumulative from data from 50 cells across experiments performed on 3 different days.

# References

1. Magzoub M., Miranker A. D. Concentration-dependent transitions govern the subcellular localization of islet amyloid polypeptide. *FASEB J*. **26**, 1228-1238 (2012).

2. Kumar S., Birol M., Schlamadinger D. E., Wojcik S. P., Rhoades E., Miranker A. D. Foldamer-mediated manipulation of a pre-amyloid toxin. *Nat. Commun.* **7**, 11412 (2016).

3. Kumar S., Birol M., Miranker A. D. Foldamer scaffolds suggest distinct structures are associated with alternative gains-of-function in a preamyloid toxin. *Chem. Commun.* **52**, 6391-6394 (2016).
